# Engagement of immune effector cells by trastuzumab induces HER2/ERBB2 downregulation in cancer cells through STAT1 activation

**DOI:** 10.1186/bcr3637

**Published:** 2014-04-02

**Authors:** Yun Shi, Xuejun Fan, Weixu Meng, Hui Deng, Ningyan Zhang, Zhiqiang An

**Affiliations:** 1 Texas Therapeutics Institute, Brown Foundation Institute of Molecular Medicine, University of Texas Health Science Center at Houston, 1825 Pressler Street, Houston, TX 77030, USA; 2 Department of Clinical Immunology and Clinical Microbiology, College of Pharmacy, the Third Military Medical University, 29 Gaotanyan Main Street, Chongqing 400038, China

## Abstract

**Introduction:**

Trastuzumab has been widely used for the treatment of human epidermal growth factor receptor 2 (HER2) overexpressing breast cancer for more than a decade. However, reports on the involvement of HER2 downregulation in trastuzumab’s mechanism of action are inconsistent. The aim of this study is to investigate if the dependence of trastuzumab-mediated cancer cell HER2 downregulation on immune effector cells represents a novel mechanism of action for trastuzumab.

**Methods:**

HER2 expression was evaluated by Western blotting, flow cytometry, and real-time polymerase chain reaction (PCR) in cell lysates from co-cultures of multiple cancer cell lines with peripheral blood mononuclear cells (PBMCs) in the presence or absence of trastuzumab. The engagement of immune cells by trastuzumab through Fc gamma receptors (FcγRs) was tested using three trastuzumab variants with compromised or no Fc (fragment crystallizable) functions and FcγRs blocking experiments. The engagement of immune cells by trastuzumab in HER2 downregulation was also evaluated in *in vivo* mouse xenograft tumor models.

**Results:**

HER2 downregulation of cancer cells by trastuzumab occurred only when trastuzumab was actively engaged with immune cells and cancer cells, as demonstrated consistently in co-cultures of cancer cell lines with PBMCs and *in vivo* mouse xenograft tumor models. We further demonstrated that HER2 downregulation in cancer cells by immune-cell-engaged trastuzumab was at the transcriptional level, not through the HER2 degradation pathway. Activation of signal transducer and activator of transcription 1 (STAT1) in cancer cells by the increased interferon gamma (IFN-γ) production in immune cells played an important role in downregulating HER2 in cancer cells upon engagement of immune cells by trastuzumab. Furthermore, HER2 downregulation in cancer cells induced by trastuzumab engagement of immune cells was correlated with the antibody’s antitumor efficacy *in vivo*.

**Conclusions:**

This study reveals that engagement of immune effector cells by trastuzumab induces HER2 downregulation in HER2-expressing cancer cells, which represents a new function of immune cells in trastuzumab-mediated antitumor efficacy and serves as a novel mechanism of action for trastuzumab. Our results imply that HER2 downregulation in cancer cells treated by trastuzumab may predict active engagement of immune effector cells in tumor microenvironment.

## Introduction

Human epidermal growth factor receptor 2 (HER2/ERBB2) is a proto-oncogene and high expression of HER2 is associated with poor prognosis in several types of cancer including breast cancer [1,2]. Downregulation of HER2 expression can suppress the cell-transforming phenotype induced by the oncogene and could be an effective way to control HER2-overexpressing tumor growth and subsequent metastasis [3,4].

Trastuzumab is a HER2-targeting monoclonal antibody for the treatment of HER2- overexpressing breast cancer [5]. After more than a decade of clinical use, mechanisms of action of trastuzumab are still not well understood, which poses significant challenges to overcome the widespread resistance to the therapy. Multiple modes of action have been proposed for trastuzumab and they can be largely classified into two categories. One class belongs to the cytostatic effects through direct interaction with HER2 on cancer cells by the Fab (antigen-binding fragment) region of the antibody, such as suppression of HER2 downstream signaling [6,7], inhibition of HER2 extracellular domain shedding [8], blocking ligand-independent HER2/HER3 heterodimerization [9]. HER2 downregulation was also proposed as a mechanism of action of trastuzumab, but it remains controversial [10,11]. Trastuzumab-mediated HER2 downregulation was shown in high HER2-expressing cancer cells in some of the earlier reports [12,13], but several studies with HER2-overexpressing cancer cells showed no HER2 downregulation by trastuzumab treatment [14-16]. There also was no HER2 reduction observed in breast tumor with HER2 overexpression in patients undergoing trastuzumab treatment [17,18]. Currently, there is no clear mechanistic explanation for these contradictory results regarding trastuzumab-mediated HER2 downregulation. The other category of mechanisms of action is mediated by the Fc (crystallizable fragment) portion of the antibody, such as antibody-dependent cellular cytotoxicity (ADCC), which is a result of engaging immune cells especially natural killer (NK) cells through the Fc portion of the antibody [19-22]. More recent evidences also point to a pivotal role of adaptive immune system in the mechanism of action of trastuzumab, such as activating specific CD8^+^ T-cell immunity [23,24]. However, the extensive role of immune responses in the mechanism of action of trastuzumab is still not fully understood.

Our previous work showed that trastuzumab treatment of cancer cells alone did not downregulate HER2 level *in vitro*, but we detected HER2 downregulation in mouse xenograft tumors when treated with trastuzumab [25], suggesting that immune cells might play a role in the HER2 downregulation by trastuzumab *in vivo*. In this study, we demonstrated that trastuzumab can induce HER2 downregulation only when it is actively engaged with immune effector cells through its Fc region. This study reveals a new role of immune cells on HER2 downregulation in response to trastuzumab treatment, which serves as a new mechanism of action of trastuzumab.

## Methods

### Cancer cells and reagents

Cancer cell lines BT474, SKBr-3, and SKOV-3 were obtained from the American Tissue Culture Collection (ATCC, Manassas, VA, USA). MCF-7/HER2 is a stable MCF-7 cell line overexpressing HER2 [26]. RPMI1640 media and fetal bovine serum (FBS) were from Invitrogen (Carlsbad, CA, USA) and penicillin streptomycin was from Sigma-Aldrich (St. Louis, MO, USA). Reagent antibodies used for assays were purchased from commercial sources as indicated. Trastuzumab was purchased from a specialty pharmacy and single hinge cleaved trastuzumab (scIgG-T) and isotype control monoclonal antibody (human immunoglobulin G1 (IgG1)) were prepared as previously described [27]. Small molecule inhibitors, MG132, chloroquine, and fludarabine were from Sigma-Aldrich.

### Co-culture of cancer cells with PBMCs

Cancer cells (1 × 10^5^) were pre-seeded in a 24-well plate (Corning, Tewksbury, MA, USA) overnight in RPMI1640 media supplemented with 10% FBS at 37°C. Human peripheral blood mononuclear cells (PBMCs) were isolated from whole blood of healthy donors (Gulf Coast Blood Center, Houston, TX, USA) using lymphocyte separation media (Cellgro, Manassas, VA, USA). The isolated PBMCs (effector cells, E) were added into cancer cells (target cells, T) at a ratio of E:T = 10:1 and cultured at 37°C, 5% CO_2_ as indicated for all treatment conditions. Conditioned media (CM) were collected after 48 h co-culture and used for study with CMs or analysis of cytokines and chemokines. PBMCs were removed from cancer cells and washed with phosphate-buffered saline (PBS) twice. Cancer cells were lysed with RIPA buffer containing proteinase inhibitor cocktails (Calbiochem, San Diego, CA, USA) for Western blotting (WB). For the Fc gamma receptor (FcγR) blocking study, PBMCs were pretreated with human isotype IgG (10 μg/ml) for 1 h and the FcγR-blocked PBMCs were then added to cancer cells in the presence or absence of trastuzumab (5 μg/ml) as described above.

### Co-cultures in transwell plate

Cancer cells were cultured in the lower chamber in the presence or absence of trastuzumab and PBMCs were added to the upper chamber using the 0.4 μm transwell insert (Corning). After 48 h co-culturing, PBMCs were removed from top chamber and cancer cells were prepared for WB analysis.

### Western blotting

Cancer cell lysates or tumor tissue lysates were subjected to SDS-PAGE separation on 10% gels (Bio-Rad Laboratories, Hercules, CA, USA) and proteins were transferred to a nitrocellulose membrane and immunoblotted with primary antibodies for anti-HER2 (Epitomics, Burlingame, CA, USA), anti-signal transducer and activator of transcription 1 (STAT1), anti-phosphorylated pSTAT1 (Cell Signaling Technology, Danvers, MA, USA), and anti-β-actin antibody (Santa Cruz Biotechnology, Santa Cruz, CA, USA) and detected with horseradish peroxidase (HRP)-conjugated secondary antibody. All WB images were captured and quantified using a FluorChem M imager (Cell BioSciences, Santa Clara, CA, USA) after adding HyGLO™ Quick Spray Chemiluminescent HRP substrate (Denville Scientific Inc., South Plainfield, NJ, USA).

### Flow cytometry

Cancer cells were detached from cell culture plates using a nonenzymatic solution with EDTA (Invitrogen) and stained with FITC-anti-HER2 antibody (BD Biosciences, San Jose, CA, USA). Briefly, 5 × 10^5^ cells were dispensed in 100 μl aliquots and stained with FITC-anti-HER2 antibodies for 30 min at 4°C. After washing with PBS buffer, cells were analyzed for mean fluorescence intensity (MFI) using a guava easyCyte HT instrument based on the manufacturer’s instructions (Millipore, Danvers, MA, USA). Data were analyzed by the FlowJo software (Tree Star Inc., Ashland, OR, USA) and light scatter characteristics were used to gate tumor cells for the analysis.

### Real-time quantitative PCR (qPCR)

Total RNA was extracted using Trizol. Two micrograms of total RNA was transcribed into cDNA using the SuperScript™ III First-Strand Synthesis System (Invitrogen). qPCR was performed on a CFX96 Touch™ real-time PCR detection system (Bio-Rad Laboratories) using the SYBR green method based on the manufacturer’s manual. Gene-specific primers are listed in Table S1 in Additional file 1. Gene expression was normalized to GAPDH and the relative gene expression was calculated using the 2^-ΔΔCt^ method [28].

### Sorting of NK, monocytes, T cells, and B cells by flow cytometry

Human PBMCs were stained with PerCP-Cy5.5-conjugated CD56, Alexa Fluor 700-conjugated CD14, and APC-Cy7-conjugated CD3 (BD Biosciences). The stained cells were then sorted by the BD FACSAria™ II flow cytometer. NK cells were collected as CD3^−^ CD14^−^ CD56^+^ cells, monocytes as CD3^−^ CD56^−^ CD14^+^, and T cells as CD3^+^ CD14^−^ CD56^−^. Similar to the co-culture study with PBMCs, the sorted immune effector cells were co-cultured with SKOV-3 cancer cells at E:T = 5:1 in the presence or absence of trastuzumab (5 μg/ml) for 48 h. Immune cells were removed and cancer cells were lysed for detecting HER2 expression by WB.

### Mouse breast cancer xenograft tumor model

Mouse tumor xenograft studies were carried out in accordance with the animal care and use guidelines, and the protocol was approved by the Animal Welfare Committee of the University of Texas Health Science Center at Houston (HSC-AWC-10-128). HER2 overexpressing BT474 and MCF7/HER2 breast cancer cells were implanted into immunodeficient nu/nu mice obtained from Charles River Laboratories (Wilmington, MA, USA) and treated with trastuzumab as previously described [25]. Tumor size was measured using a Vernier scale caliper and tumor growth inhibition was calculated as ‘(1 - mean tumor volume of the test group/mean tumor volume of the control group) × 100’. Fresh tumor tissues were collected by snap freeze in liquid nitrogen for WB analysis and 10% formaldehyde-fixed tumor tissues were used for HER2 detection by immunohistochemistry (IHC).

### IHC detection of HER2

HER2 levels in xenograft tumor tissues after trastuzumab treatment were detected using a primary anti-HER2 antibody from Epitomics. An HRP polymer system (Dako, Glostrup, Denmark) was used for tissue staining according to the manufacturer’s procedures.

### Detection of IFN-γ

Interferon gamma (IFN-γ) expression in the cell supernatants was detected by an enzyme-linked immunosorbent assay (ELISA) and both capture and detection antibodies were from R&D Systems (Minneapolis, MN, USA). Briefly, the ELISA plate was coated overnight with anti-IFN-γ capture antibody (1 μg/ml). After blocking with 5% bovine serum albumin (BSA)-PBS, diluted standard IFN-γ (ProSpec, East Brunswick, NJ, USA) and the cell lysates were added and incubated overnight, followed by a secondary anti-IFN-γ biotinylated capture antibody (0.5 μg/ml) for 2 h. After washing, the biotinylated antibody was detected by streptavidin-labeled HRP for 30 min. After washing, TMB was used as substrate to develop color and plates were read at 450 nm on a SpectraMax M4 microplate reader (Molecular Devices, Sunnyvale, CA, USA). IFN-γ concentration in the samples was derived using a standard curve.

### Statistical analysis

Where appropriate, statistical analysis was performed using Student’s *t* test. A *P* value <0.05 between treatment groups is considered significantly different. Experiments were repeated at least three times.

## Results

### HER2 downregulation in cancer cells by trastuzumab in the presence of PBMCs

**Figure 1:**
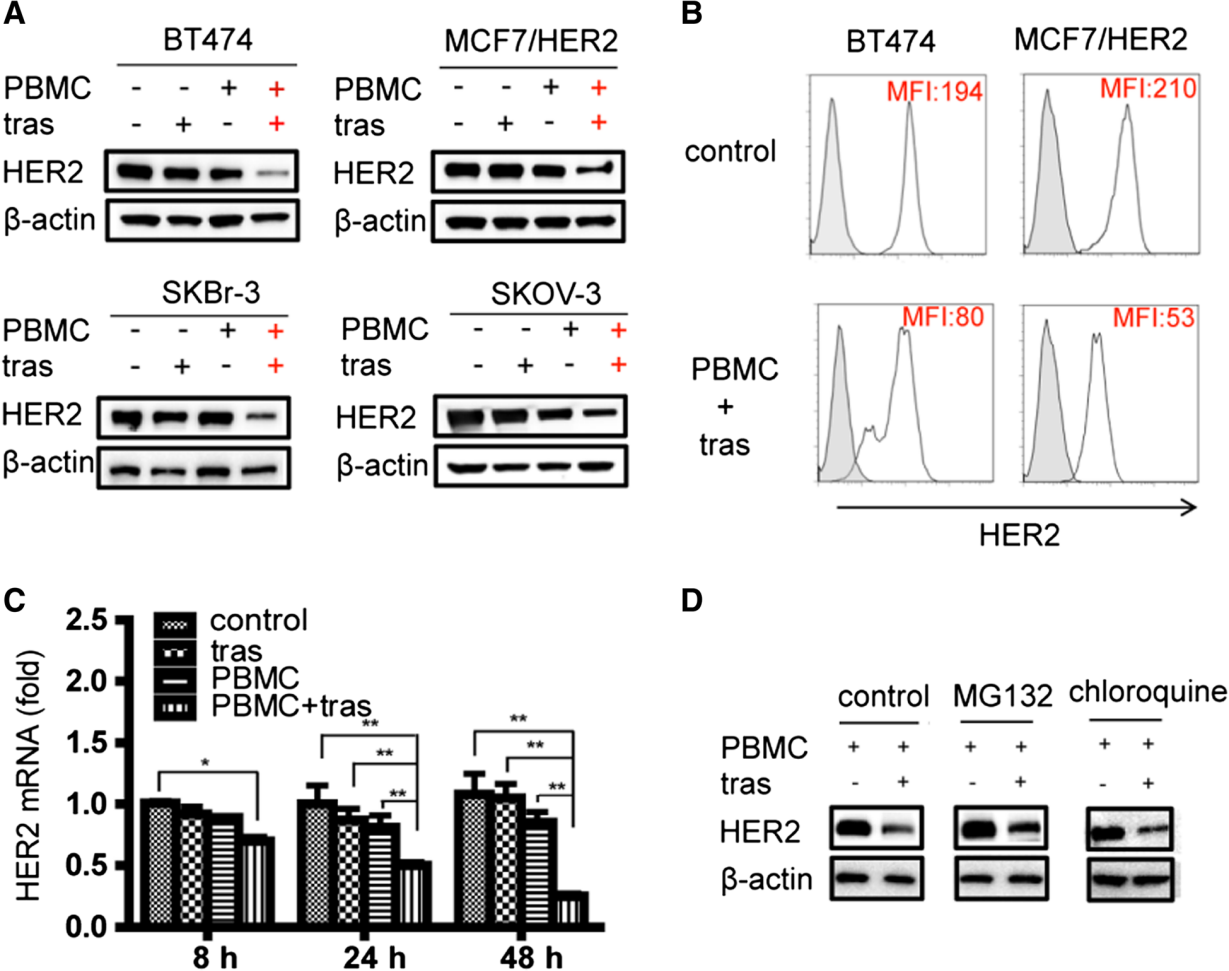
**HER2 downregulation in cancer cells by PBMCs in the presence of trastuzumab. (A)** WB detection of HER2 levels in four high HER2 cancer cells BT474, MCF7/HER2, SKBr3, and SKOV-3 cultured in different conditions for 48 h. PBMCs were added at E:T ratio = 10:1 in the presence of trastuzumab (tras) or isotype control IgG (5 μg/ml). Cell lysates were loaded 20 μg per well and β-actin was detected as a loading control. Four treatment conditions are shown for each of the four cancer cell lines and the condition with PBMC and trastuzumab is labeled with the red +. **(B)** HER2 levels on the cell surface were detected by flow cytometry. Histograms shown in the upper panel are for cancer cells without treatment and the lower panel is for cancer cells with PBMC and trastuzumab treatment. The filled histogram is for cells stained with an isotype antibody control and the open histogram is for cells stained with an anti-HER2 antibody. The mean fluorescence intensity (MFI) of HER2 staining is shown in each histogram. **(C)** qPCR detection of HER2 mRNA levels in BT474 cancer cells at 8 h, 24 h, and 48 h posttreatment. *P <0.05; **P <0.01. **(D)** WB detection of HER2 in BT474 cells from co-culture with PBMCs in the presence or absence of trastuzumab under three conditions: no inhibitor control (left); addition of the proteasome inhibitor MG-132 (middle), and addition of the lysosome inhibitor chloroquine (right). HER2, human epidermal growth factor receptor 2; IgG, immunoglobulin G; PBMC, peripheral blood mononuclear cell; WB, Western blotting.

We previously observed that HER2 level in high HER2-expressing BT474 breast cancer cells was not affected by trastuzumab treatment *in vitro*, but HER2 was downregulated in BT474 mouse xenograft tumors treated with trastuzumab [25]. To test whether immune cells play a role in HER2 downregulation in response to trastuzumab treatment, four high HER2-expressing cancer cell lines were treated with trastuzumab in the presence or absence of human PBMCs. HER2 levels in cancer cells were detected by WB after 48 h of co-culture treatment. Treatments of cancer cells with trastuzumab or PBMCs alone had no effects on HER2 levels in cancer cells (Figure 1A). In contrast, HER2 levels in cancer cells co-cultured with PBMCs in the presence of trastuzumab were significantly reduced in all four high HER2-expressing cancer cell lines in comparison with cancer cell controls or with trastuzumab or PBMCs single treatment (Figure 1A). To test if the low HER2 levels detected in the whole cell lysates by WB in cancer cells treated with PBMCs and trastuzumab reflects HER2 downregulation on the cell surface, we measured surface HER2 levels by a flow cytometer. Consistent with HER2 levels detected in the whole cell lysates by WB, HER2 levels determined by flow cytometry were lower on the cancer cell surface with co-treatment of trastuzumab and PBMCs than that on control cells as indicated by the decreased MFI in the flow histograms (Figure 1B). To understand if HER2 downregulation mediated by trastuzumab in the presence of PBMCs is a result of reduced HER2 gene transcription, we determined HER2 mRNA levels by qPCR after different time points of co-treatment of cancer cells with human PBMCs and trastuzumab. As shown in Figure 1C, HER2 mRNA level in BT474 cells was significantly reduced when co-cultured with PBMCs in the presence of trastuzumab, while treatment of cancer cells with trastuzumab or PBMCs alone had no effects on HER2 mRNA levels. The decrease of HER2 mRNA level in BT474 cells treated with PBMCs and trastuzumab was detectable after 8 h, and the reductions reached 50% and 75% after 24 h and 48 h of treatment, respectively (Figure 1C). To study if protein stability played a role in the PBMCs and trastuzumab-mediated HER2 downregulation, we measured HER2 levels in cancer cells treated with PBMCs and trastuzumab in the presence of a proteasome inhibitor (MG132) or a lysosome inhibitor (chloroquine). As shown in Figure 1D, inhibitors of the protein degradation pathways (MG132 or chloroquine) had no effects on the HER2 downregulation mediated by PBMCs and trastuzumab co-treatment. Taken together, these results demonstrated that co-treatment of high HER2 cancer cells with PBMCs and trastuzumab led to HER2 downregulation at the transcriptional level.

### Engagement of Fc gamma receptors (FcγRs) on immune cells through trastuzumab Fc is essential for the HER2 downregulation

**Figure 2:**
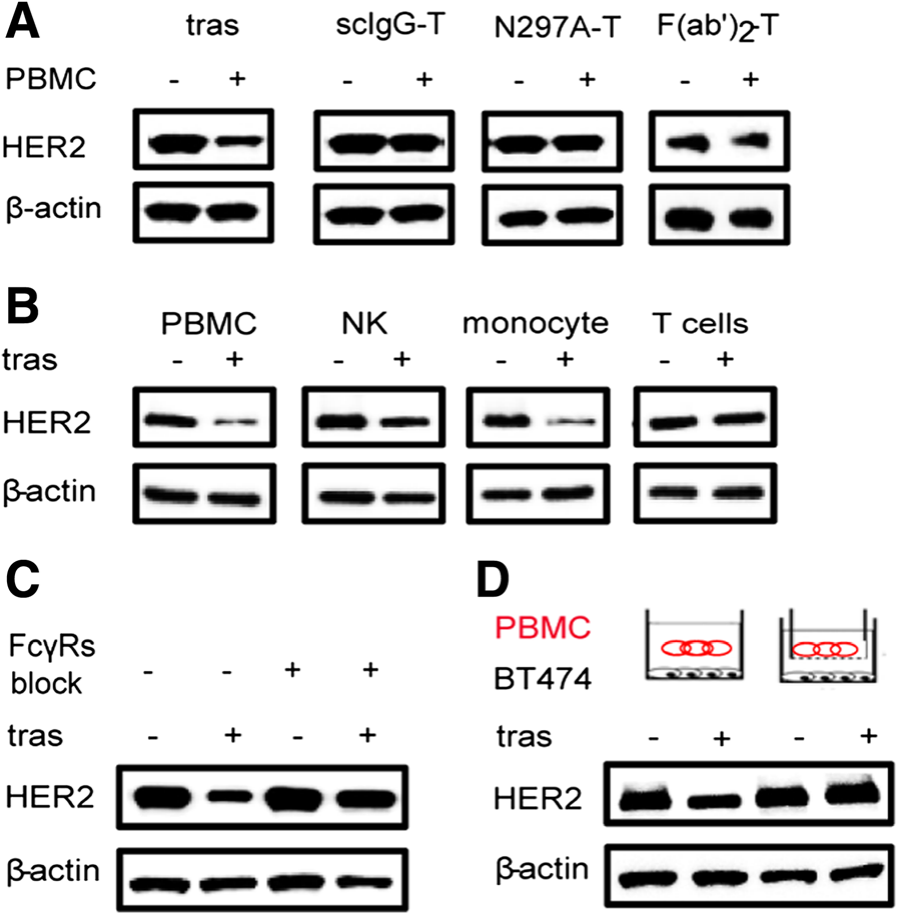
**Engagement of FcγRs on immune cells with trastuzumab Fc is required for HER2 downregulation. (A)** WB detection of HER2 in BT474 breast cancer cells with or without co-culture with PBMCs in the presence of trastuzumab, scIgG-T, N297A-T, or F(ab’)_2_-T for 48 h as labeled on the top of each panel. The same amount of protein lysates was loaded on each lane and β-actin was used as a loading control. **(B)** WB detection of HER2 in BT474 cancer cells after co-culture with NK cells, monocytes, T cells and PBMCs as labeled on each panel in the presence and absence of trastuzumab for 48 h. **(C)** PBMCs were pretreated with isotype IgG (10 μg/ml) for 1 h to block FcγRs before conducting co-culture with BT474 cancer cells in the presence or absence of trastuzumab (5 μg/ml) for 48 h. HER2 expression was detected by WB. **(D)** WB detection of HER2 downregulation in cancer cells after co-culture in transwell chambers with PBMC (the right two lanes) in the presence and absence of trastuzumab. Cancer cells co-cultured with PBMCs without the transwell chamber (the left two lanes) were used as controls. Fab, fragment, antigen binding; Fc, fragment, crystallizable; FcγR, Fc gamma receptor; HER2, human epidermal growth factor receptor 2; NK cell, natural killer cell; PBMC, peripheral blood mononuclear cell; scIgG-T, single hinge cleaved trastuzumab; WB, Western blotting.

To test whether the interaction between trastuzumab Fc and FcγRs on immune cells is required for HER2 downregulation in cancer cells, we used three variants of trastuzumab with compromised or no Fc functions [25,29,30]: the scIgG-T variant has a single proteolytic cleavage at the hinge region of trastuzumab; the N297A-T has one amino acid mutation at the position 297 (from N to A, European numbering) and lacks N-glycosylation and Fc function; and the F(ab’)_2_-T was generated by cleavage of trastuzumab Fc with the protease pepsin. Unlike the cancer cells treated with trastuzumab and PBMCs, cancer cells treated with the scIgG-T, N297A-T, or F(ab’)_2_-T in the presence of PBMCs showed no HER2 downregulation (Figure 2A). Since immune cell subtypes have different expression profiles of FcγRs (Figure S1 in Additional file 1), we isolated NK cells, monocytes, and T cells (no detectable FcγRs) from PBMCs by a flow cytometer with a cell sorter and HER2 downregulation mediated by the co-treatment of trastuzumab and different immune cell subtypes were evaluated. Similar to the cancer cells treated with PBMCs and trastuzumab, cancer cells showed HER2 downregulation after co-treatment with trastuzumab and NK cells or monocytes, but cancer cells treated with T cells and trastuzumab did not show HER2 downregulation (Figure 2B). Furthermore, we blocked trastuzumab Fc binding with FcγRs on immune cells by preincubating PBMCs with human isotype IgGs before co-culturing with cancer cells. The preblocking of FcγRs on PBMCs with isotype IgGs abolished the HER2 downregulation mediated by PBMCs in the presence of trastuzumab (Figure 2C). These data suggest that engagement of FcγRs on immune cells by trastuzumab Fc is required for HER2 downregulation in cancer cells.

To further test whether direct engagement between cancer cells and immune cells is necessary for trastuzumab to trigger HER2 downregulation, we employed a transwell co-culturing system that retained immune cells in the upper chamber without direct contact with cancer cells at the bottom of the culture system. As shown in Figure 2D, cancer cells in the transwell co-culturing condition showed no HER2 downregulation, suggesting that direct engagement of cancer cells and immune cells is important for HER2 downregulation mediated by PBMCs and trastuzumab. Collectively, these results indicate that HER2 downregulation in cancer cells requires direct interaction of the trastuzumab Fab region with the HER2 antigen on cancer cells and the Fc region with FcγRs on immune cells, which brings cancer cells and immune cells in proximity for activating the immune effector function.

### HER2 downregulation in cancer cells by the cell-free conditioned medium

**Figure 3:**
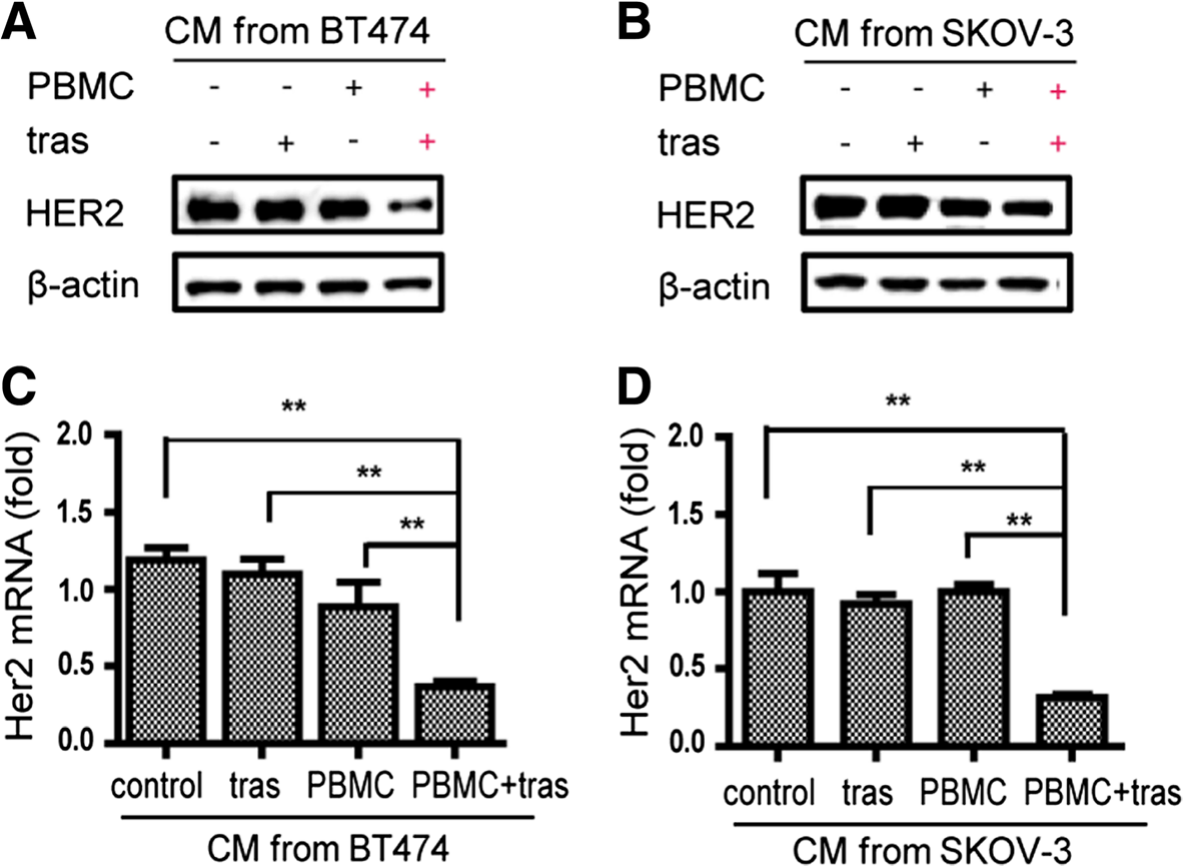
**HER2 downregulation in cancer cells by the CM from cancer cells co-cultured with PBMCs in the presence of trastuzumab.** Both BT474 **(A, C)** and SKOV-3 cells **(B, D)** were cultured for 48 h with 50% CM collected from the co-cultures of four treatment conditions: cancer cell only, cancer cells treated with trastuzumab, cancer cell co-cultured with PBMCs, and cancer cell co-cultured with PBMCs in the presence of trastuzumab. **(A-B)** WB detection of HER2 level; **(C-D)** HER2 mRNA levels detected by qPCR, **P <0.01. CM, conditioned media; HER2, human epidermal growth factor receptor 2; PBMC, peripheral blood mononuclear cell; WB, Western blotting.

Activated immune cells secrete an array of soluble effectors. To determine if cancer cell HER2 downregulation is a function of soluble effectors secreted from the activated immune cells in the presence of trastuzumab, we tested HER2 downregulation in cancer cells after culturing with the CM collected from co-cultures of cancer cells and PBMCs in the presence or absence of trastuzumab. Cancer cells were cultured in 50% (1:1 diluted with fresh media) CM that were collected from four culturing conditions: cancer cell only, cancer cells treated with trastuzumab alone, cancer cells plus PBMCs, and cancer cells plus PBMCs and trastuzumab. Similar to the results shown in Figure 1A using the cells from co-culturing conditions, the CM from the co-culture of cancer cells and PBMCs in the presence of trastuzumab also showed significant HER2 downregulation (Figure 3A, B). HER2 mRNA levels were significantly reduced in both BT474 and SKOV-3 high HER2 cancer cell lines when cultured in the CM collected from the co-cultures of the cancer cells and PBMCs plus trastuzumab, but not in CMs collected from the other conditions (Figure 3C, D). These results suggest that HER2 downregulation in cancer cells is induced by certain soluble effectors released in the CM during the co-culture of cancer cells and PBMCs in the presence of trastuzumab.

### STAT1 activation leads to HER2 downregulation in cancer cells

**Figure 4:**
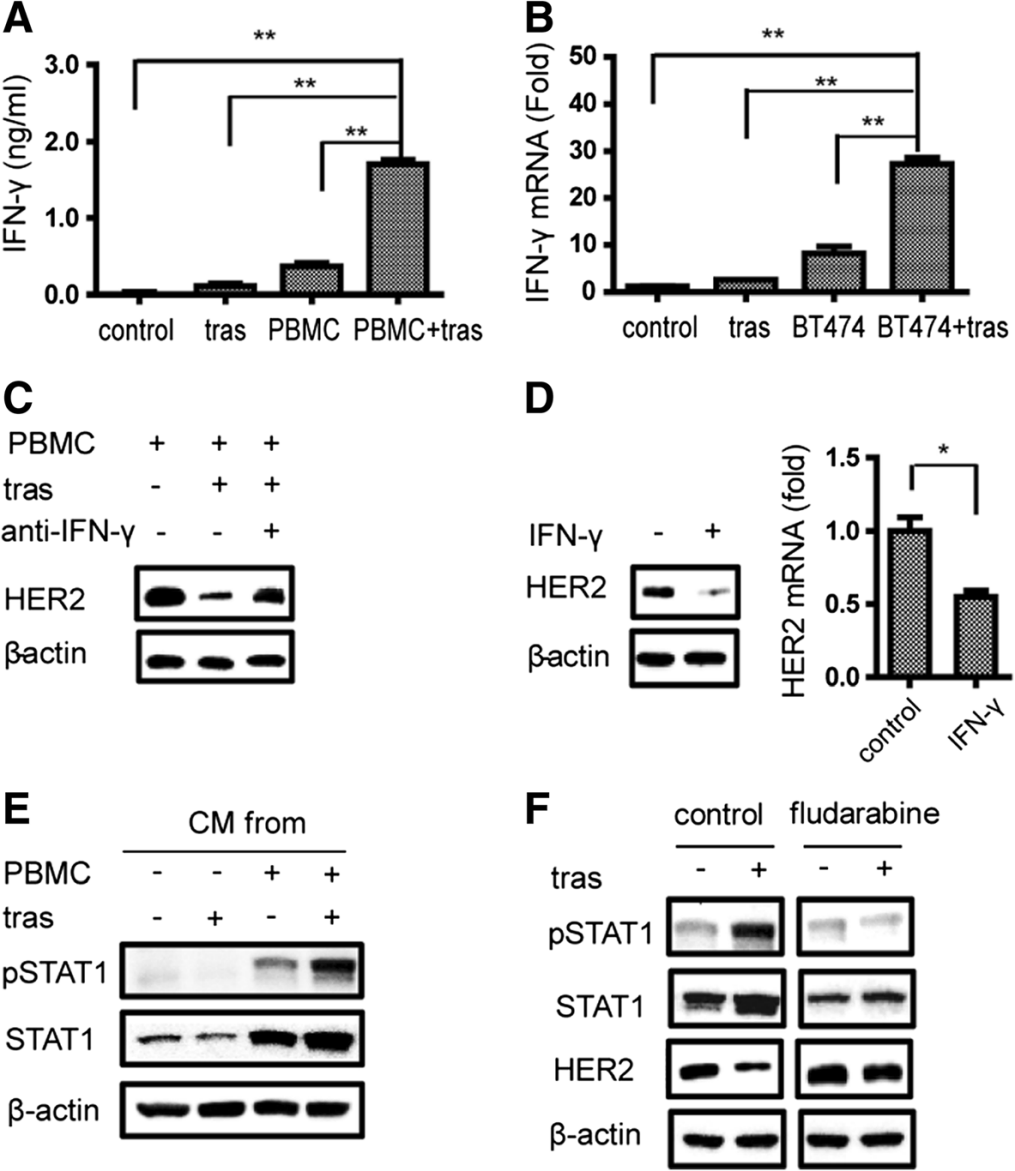
**Effects of IFN-γ and STAT1 on HER2 downregulation mediated by PBMCs in the presence of trastuzumab. (A)** IFN-γ detection in the CM by ELISA. BT474 cells were cultured for 48 h in conditions shown at the X-axis and the CM from co-culture with PBMCs in the presence of trastuzumab showed significantly higher level than the other three conditions, **P <0.01. **(B)** IFN-γ mRNA levels in PBMCs detected by qPCR. PBMCs were incubated in media (control), with trastuzumab (tras), with formaldehyde-inactivated BT474 cells (BT474), and inactivated BT474 cells plus trastuzumab (BT474 + tras) for 24 h before isolation of total RNA for qPCR. **P <0.01. **(C)** WB detection of HER2 in BT474 cells with and without the addition of an IFN-γ-neutralizing antibody (10 μg/ml) for 48 h as indicated on the top of each lane. **(D)** Effects of IFN-γ (100 ng/ml) treatment on HER2 expression in BT474 cells. WB detection is shown on the left and mRNA level by qPCR is shown in the bar graph on the right. *P <0.05. **(E)** WB detection of total pSTAT1 and STAT1 in BT474 cells after culturing with CM collected from four culture conditions as shown on the top of each lane: cancer cells only, cancer cells treated with trastuzumab, cancer cells plus PBMCs, and cancer cells plus PBMCs and trastuzumab. The β-actin band serves as a loading control. **(F)** Effects of fludarabine on pSTAT1, total STAT1, and HER2 levels as detected by WB. BT474 cells were cultured in 50% CM collected from BT474 cells co-cultured with PBMCs ± trastuzumab, in the presence or absence of fludarabine (100 μM) as indicated on the top. CM, conditioned media; ELISA, enzyme-linked immunosorbent assay; HER2, human epidermal growth factor receptor 2; IFN-γ, interferon gamma; PBMC, peripheral blood mononuclear cell; (p)STAT1, (phosphorylated) signal transducer and activator of transcription 1; WB, Western blotting.

To investigate the soluble effectors in the CM that resulted in the HER2 downregulation in cancer cells, we profiled cytokines and chemokines in the CMs using a cytokine array. Levels of cytokines and chemokines in the CM from the co-culture of BT474 cells with PBMCs in the presence of trastuzumab were compared to that in the CM from the co-culture of BT474 cells with PBMCs only. A panel of cytokines and chemokines including IFN-γ showed higher levels in the CM from the co-culture of BT474 cells with PBMCs in the presence of trastuzumab than that in the absence of trastuzumab (Figure S2 in Additional file 1). Since IFN-γ has been shown to exhibit inhibitory effects on HER2 expression in cancer cells *in vitro*[31], we investigated whether the increased IFN-γ in the CM from the co-treatment of cancer cells with PBMCs and trastuzumab has a role in the cancer cell HER2 downregulation. IFN- γ production under the different treatment conditions was quantified by an ELISA. As shown in Figure 4A, the co-culture of BT474 cells with PBMCs in the presence of trastuzumab produced significantly more IFN-γ than the other culture conditions. Culture media from BT474 cells only or BT474 cells with trastuzumab had no detectable IFN-γ.

To determine if the increased IFN-γ expression was a result of activation of PBMCs after their engagement with cancer cells through trastuzumab Fc, we first inactivated cancer cells with 4% paraformaldehyde (PFA); the inactivated BT474 cells were then co-cultured with PBMCs in the presence or absence of trastuzumab for 24 h; and IFN-γ mRNA levels in live PBMCs were determined by qPCR. As shown in Figure 4B, the PBMCs in the presence of both trastuzumab and the inactivated cancer cells (trastuzumab-coated cancer cells) had significantly higher IFN-γ mRNA than that in the other three conditions: PBMCs only, PBMCs with trastuzumab alone, or PBMCs with the inactivated cancer cell alone (Figure 4B). The results indicate that PBMCs secrete more IFN-γ upon activation in the presence of trastuzumab-coated cancer cells. To determine if the elevated IFN-γ contributes to HER2 downregulation in cancer cells, we used an IFN-γ-neutralizing antibody to block IFN-γ function. As shown in Figure 4C, HER2 downregulation was rescued by the IFN-γ-neutralizing antibody, while addition of exogenous IFN-γ in the cancer cell cultures induced HER2 downregulation at both the protein level and the mRNA level (Figure 4D).

Among the multiple signaling pathways regulated by IFN-γ, signal transducer and activator of transcription 1 (STAT1) has been reported to be associated with HER2 downregulation [31]. We measured STAT1 expression and activation in BT474 cells cultured in the CM collected from the co-culture of BT474 and PBMCs in the presence of trastuzumab. Total STAT1 protein and pSTAT1 (Tyr701) were significantly increased in BT474 cells treated with the CM collected from co-treatment with PBMCs and trastuzumab, when compared with the cells cultured with CMs from the other treatments (Figure 4E). Addition of fludarabine, an inhibitor of STAT1, reduced levels of both pSTAT1 and total STAT1 and blocked HER2 downregulation in cancer cells cultured with the CM from the co-treatment with PBMCs and trastuzumab (Figure 4F). Collectively, these results suggest that elevated IFN-γ expression by activated PBMCs and STAT1 activation in cancer cells play an important role in HER2 downregulation in cancer cells by the co-treatment of PBMCs and trastuzumab.

### Immune cell engagement by trastuzumab mediates HER2 downregulation and antitumor efficacy *in vivo*

**Figure 5:**
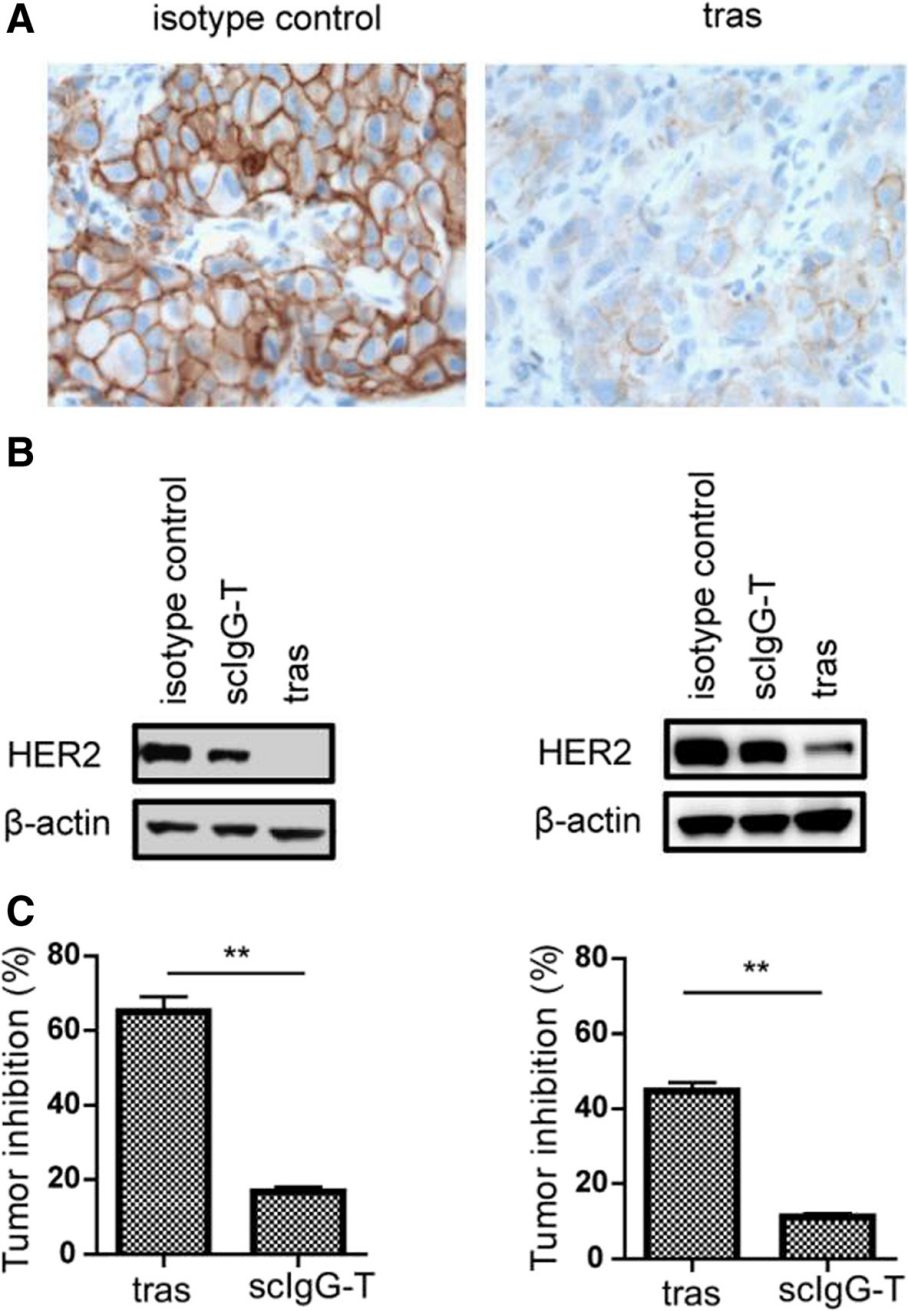
**HER2 downregulation and tumor inhibition by trastuzumab in mouse xenograft tumor models.** Tumors from different treatment groups (n = 3) were collected one day after last administration of the antibody. **(A)** Formaldehyde-fixed BT474 tumor tissues were used for IHC detection of HER2. **(B)** The same amounts of tumor lysates were loaded on SDS-PAGE and HER2 was detected by WB. **(C)** Tumor inhibition after four weekly treatments with trastuzumab (tras) or the variant scIgG-T. The left bar graph is from the BT474 tumor model and the right graph is from the MCF7/HER2 tumor model. Percentage of tumor inhibition was calculated, **P <0.01. HER2, human epidermal growth factor receptor 2; IHC, immunohistochemistry; scIgG-T, single hinge cleaved trastuzumab; WB, Western blotting.

As expected, HER2 downregulation in cancer cells by trastuzumab engagement of immune cells was correlated with the inhibition of cancer cell proliferation (Figure S3 in Additional file 1). To understand whether active engagement of immune cells *in vivo* by trastuzumab can also mediate HER2 downregulation, we used a mouse xenograft tumor model that we showed immune cell engagement by trastuzumab previously [25]. Tumor-bearing mice were treated with trastuzumab or scIgG-T (with a compromised Fc) weekly at 5 mg/kg for four weeks and an isotype IgG was used as nontreatment control. IHC detection of HER2 level in the BT474 tumor tissue treated with trastuzumab showed significant downregulation in comparison with that in tumor tissue treated with isotype control antibody (Figure 5A). In the Western blotting studies, HER2 levels in the residue tumor tissues after trastuzumab treatment were barely detectable by WB, while HER2 levels remained high in tumors treated with scIgG-T or isotype control antibodies in both BT474 and MCF7/HER2 tumor models (Figure 5B). As scIgG-T has less capability of FcγRs engagement in comparison with trastuzumab, the reduced HER2 downregulation by scIgG-T supports the notion that HER2 downregulation in cancer cells requires active engagement of immune cells by the antibody Fc. Treatment with scIgG-T antibody also showed reduced antitumor efficacy than trastuzumab in both BT474 and MCF7/HER2 xenograft tumor models (Figure 5C). The correlation between less HER2 downregulation and decreased anticancer efficacy by scIgG-T treatment *in vivo* suggests that HER2 downregulation directly contributes to the antitumor efficacy of trastuzumab.

## Discussion

Although targeting HER2 by trastuzumab has proven an effective strategy for treatment of breast cancer with HER2 overexpression, widespread resistance to the therapy poses significant challenges in the clinic. Lack of full understanding on the mechanisms of action for trastuzumab is one of major obstacles for overcoming the resistance. Among the many proposed mechanisms of action for trastuzumab, it is controversial whether HER2 downregulation contributes to trastuzumab efficacy [10,11]. Some studies reported HER2 downregulation by trastuzumab in high HER2 cancer cell cultures [12,13], while some clinical studies showed no reduction in tumor HER2 expression in patients undergoing trastuzumab treatment [17,18]. Our results showed the two-sided effect of trastuzumab on HER2 downregulation depending on the engagement of immune cells. The lack of HER2 downregulation induced by trastuzumab observed in previous studies may be the result of low or absence of active immune effector cells in the cell culture conditions *in vitro* or tumor microenvironments *in vivo*. This study demonstrated that trastuzumab treatment alone did not downregulate HER2 levels in cancer cells *in vitro*, but HER2 levels were downregulated when immune effector cells were engaged with trastuzumab. Our results suggest that immune cells play crucial roles in the trastuzumab-induced HER2 downregulation. This was further supported by the *in vivo* xenograft studies that a functional Fc was required for trastuzumab to induce HER2 downregulation, as the trastuzumab variant scIgG-T was unable to mediate HER2 downregulation due to the lack of FcγR engagement on immune cells in the tumor microenvironment.

Formation of the cancer cell/trastuzumab/immune cell complex is necessary for trastuzumab-mediated HER2 downregulation. This notion is supported by three pieces of evidence presented in this study: 1) co-culturing cancer cells with immune cells alone did not drive HER2 downregulation in cancer cells; 2) physical separation of cancer cells and PBMCs in the transwell assay abolished the trastuzumab-mediated HER2 downregulation in cancer cells; and 3) trastuzumab variants with compromised Fc function did not induce HER2 downregulation in cancer cells.

It is well established that ADCC-induced cytotoxic cancer cell killing is a function of direct immune effector cell engagement with cancer cells mediated by trastuzumab [19,21,22]. It is important to note that the role of immune cells in HER2 downregulation of cancer cells mediated by trastuzumab results in a cytostatic inhibition of cancer cell growth, which is a new function of immune cells in trastuzumab efficacy. In our co-culture studies, HER2 downregulation was detected under an effector to target (E:T) ratio of 10:1, but such E:T ratio could not trigger ADCC activity *in vitro*. In addition, HER2 downregulation was detectable after 48 h of co-culturing while ADCC happened much earlier (<24 h). These results suggest that it is less likely that ADCC contributed to the HER2 downregulation by selectively killing off cancer cells with higher HER2 expression. Therefore, HER2 downregulation in the presence of trastuzumab represents a new mode of action for trastuzumab through engagement of immune cells.

Strong correlation of elevated IFN-γ expression with HER2 downregulation indicates that IFN-γ may play an important role in HER2 downregulation upon trastuzumab engagement of immune cells. Supplementing of IFN-γ in cancer cell cultures can downregulate HER2 and blocking IFN-γ in the co-culture system could rescue the HER2 downregulation induced by PBMC and trastuzumab (Figure 4) and IFN-γ played a critical role in the anticancer efficacy of an anti-HER2 mAb therapy [32]. In addition to IFN-γ, several other cytokines (such as interleukin (IL)-1β, IL-3, and IL-12p70) also showed increased production in the co-culture of cancer cells and immune cells in the presence of trastuzumab (Figure S2 in Additional file 1). Whether those cytokines also play a role in HER2 downregulation needs further investigation.

Activation of STAT1 by IFN-γ has been implicated in HER2 downregulation [31]. We showed in this study that total STAT1 protein and phosphorylation of STAT1 were increased in cancer cells with HER2 downregulation. In addition, inhibition of STAT1 with fludarabine blocked the HER2 downregulation mediated by immune cells in the presence of trastuzumab. Taken together, we propose that engagement of immune cells and cancer cells by trastuzumab triggers the release of IFN-γ, which activates STAT1-mediated transcriptional downregulation of HER2 in cancer cells.

## Conclusions

This study demonstrates that active engagement of immune effector cells and high HER2-expressing cancer cells by trastuzumab induces HER2 downregulation through the increased secretion of IFN-γ by immune cells and activation of STAT1 in cancer cells. The dependence of trastuzumab-mediated HER2 downregulation on immune effector cells represents a novel mechanism of action of trastuzumab. More importantly, our results imply that HER2 downregulation in cancer cells treated by trastuzumab may predict active engagement of immune effector cells in tumor microenvironment. Further investigation on the correlation between trastuzumab-mediated immune effector function and HER2 downregulation in patients treated with trastuzumab should help us to better understand the role of immune modulation in the anticancer efficacy of trastuzumab.

## Abbreviations

ADCC: antibody-dependent cellular cytotoxicity; ATCC: American Type Culture Collection; BSA: bovine serum albumin; CM: conditioned media; ELISA: enzyme-linked immunosorbent assay; Fab: fragment, antigen binding; FBS: fetal bovine serum; Fc: fragment, crystallizable; FcγR: Fc gamma receptor; HER2: human epidermal growth factor receptor 2; HER3: human epidermal growth factor receptor 3; HRP: horseradish peroxidase; IFN-γ: interferon gamma; IgG: immunoglobulin G; IHC: immunohistochemistry; IL: interleukin; MFI: mean fluorescence intensity; NK cell: natural killer cell; PBMC: peripheral blood mononuclear cell; PCR: polymerase chain reaction; PBS: phosphate-buffered saline; PFA: paraformaldehyde; qPCR: quantitative real-time PCR; RPMI 1640: cell culture media developed at Roswell Park Memorial Institute; scIgG-T: single hinge cleaved trastuzumab; SDS-PAGE: sodium dodecyl sulfate-polyacrylamide gel electrophoresis; STAT1: signal transducer and activator of transcription 1; WB: Western blotting.

## Competing interests

The authors declare no competing interests.

## Authors’ contributions

YS participated in the study design, Western blotting assays, flow cytometry, and wrote the manuscript. XF conducted mouse tumor xenograft studies, analyzed data, and contributed to the drafting of the manuscript. WM contributed to the real-time PCR assay, interpreted data, and contributed to the drafting of the manuscript. HD conducted the cell culture, cell proliferation assay, analyzed data, and contributed to the drafting of the manuscript. ZA conceived the study and wrote the manuscript. NZ designed experiments, interpreted data, and wrote the manuscript. All authors read and approved the final manuscript.

## Supplementary Material

Additional file 1: Figure S1 Fc gamma receptor (FcγR) expression on monocytes and natural killer (NK) cells. **Figure S2.** Cytokine and chemokine profiling in BT474 cells treated with peripheral blood mononuclear cells (PBMCs) plus trastuzumab. **Figure S3.** Human epidermal growth factor receptor 2 (HER2) downregulation in cancer cells by trastuzumab engagement of immune cells resulted in inhibition of cancer cell proliferation. **Table S1.** Primers for real‒time PCR. Click here for file
