# Supplementary material for: Engagement of immune effector cells by trastuzumab induces HER2/ERBB2 downregulation in cancer cells through STAT1 activation
Source: Breast Cancer Res. 2014 Apr 2;16(2):R33. doi: 10.1186/bcr3637 (PMC4053225; doi:10.1186/bcr3637)
Supplement: Additional file 1: Figure S1 — Fc gamma receptor (FcγR) expression on monocytes and natural killer (NK) cells. Figure S2. Cytokine and chemokine profiling in BT474 cells treated with peripheral blood mononuclear cells (PBMCs) plus trastuzumab. Figure S3. Human epidermal growth factor receptor 2 (HER2) downregulation in cancer cells by trastuzumab engagement of immune cells resulted in inhibition of cancer cell proliferation. Table S1. Primers for real‒time PCR. [file bcr3637-S1.docx]

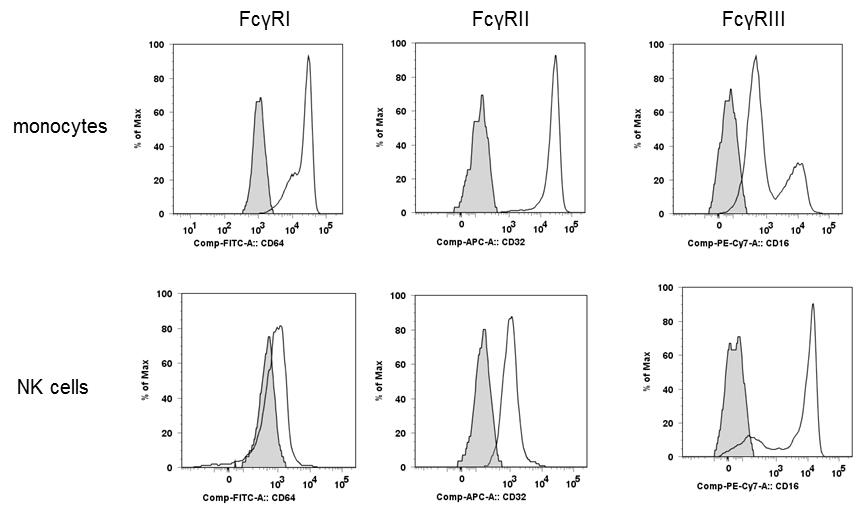


Additional file 1: Figure S1. FcγRs expression on monocytes and NK cells. PBMC were stained with Alexa Fluor 700-anti-CD14, PerCP-Cy5.5-anti-CD56, FITC-anti-CD64, APC-anti-CD32, PE-Cy7-anti-CD16 and the cells were analyzed with BD FACSAria II. CD14^+^ cells were gated as monocytes and CD56^+^ cells were gated as NK cells. Each FcγR expression on monocytes or NK cells was analyzed. The grey histogram represents isotype control and the overlayed light histogram represents the corresponding staining with anti-FcγRs.


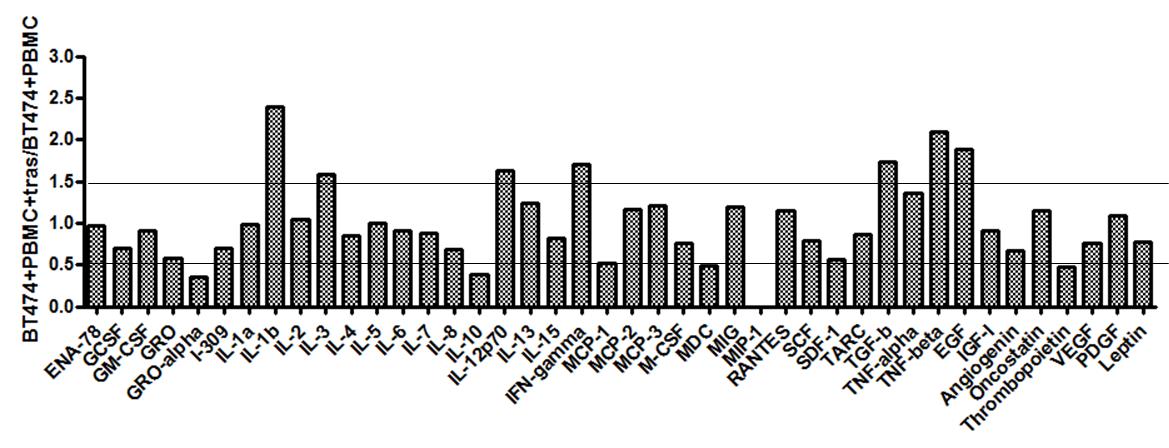


Additional file 1: Figure 2. Cytokines and chemokines profiling in BT474 cells treated with PBMC plus trastuzumab. BT474 cells were cultured with PBMC with/without trastuzumab for 48 h. The supernatants were collected and the cytokines production was measured using RayBio R Human cytokine antibody array G-3 according to the manufacture`s instruction. The increase of 1.5 fold or decrease of 0.5 fold are considered as significant change.


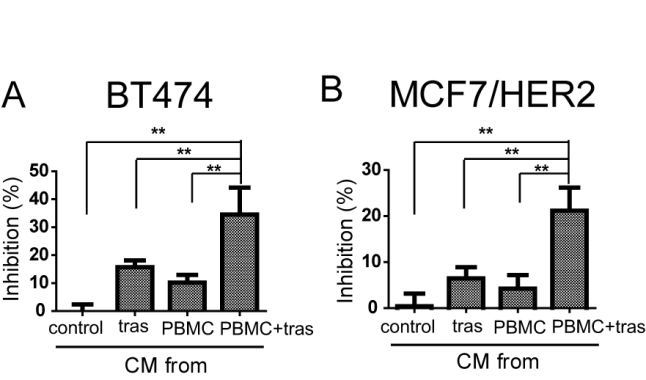


Additional file 1: Figure 3.  HER2 downregulation in cancer cells by trastuzumab engagement of immune cells resulted in inhibition of cancer cell proliferation. **(A)** BT474 and **(B)** MFC7/HER2 cells were cultured with conditioned media (CM) from the same cancer cells with or without PBMCs, and in the presence or absence of trastuzumab for 72 h. Cell proliferation was detected by Alarma blue.

Additional file 1: **Table S1. Primers for real‐time PCR**

| Genes | Forward | Reverse |
| --- | --- | --- |
| \| GAPDH \| 5-cggagtcaacggatttggtcgtat-3 \| 5-agccttctccatggtggtgaagac-3 \| \| --- \| --- \| --- \| | 5´-cggagtcaacggatttggtcgtat-3´ | 5´-agccttctccatggtggtgaagac-3´ |
| \| HER2 \| 5_-cgggagatccctgacctgctggaa-3 \| 5-ctgctggggtaccagatactcctc-3 \| \| --- \| --- \| --- \| | 5´-cgggagatccctgacctgctggaa-3´ | 5´-ctgctggggtaccagatactcctc-3´ |
| IFN-γ | 5´-GGCTTTTCAGCTCTGCATCG-3´ | 5´-TCTGTCACTCTCCTCTTTCCAA-3´ |
